# Astrocytes and Inflammatory T Helper Cells: A Dangerous Liaison in Multiple Sclerosis

**DOI:** 10.3389/fimmu.2022.824411

**Published:** 2022-02-08

**Authors:** Martina Kunkl, Carola Amormino, Valentina Tedeschi, Maria Teresa Fiorillo, Loretta Tuosto

**Affiliations:** ^1^ Department of Biology and Biotechnology Charles Darwin, Sapienza University, Rome, Italy; ^2^ Laboratory Affiliated to Istituto Pasteur Italia-Fondazione Cenci Bolognetti, Sapienza University, Rome, Italy

**Keywords:** multiple sclerosis, astrocytes, Th cells, inflammation, demyelination

## Abstract

Multiple Sclerosis (MS) is a neurodegenerative autoimmune disorder of the central nervous system (CNS) characterized by the recruitment of self-reactive T lymphocytes, mainly inflammatory T helper (Th) cell subsets. Once recruited within the CNS, inflammatory Th cells produce several inflammatory cytokines and chemokines that activate resident glial cells, thus contributing to the breakdown of blood-brain barrier (BBB), demyelination and axonal loss. Astrocytes are recognized as key players of MS immunopathology, which respond to Th cell-defining cytokines by acquiring a reactive phenotype that amplify neuroinflammation into the CNS and contribute to MS progression. In this review, we summarize current knowledge of the astrocytic changes and behaviour in both MS and experimental autoimmune encephalomyelitis (EAE), and the contribution of pathogenic Th1, Th17 and Th1-like Th17 cell subsets, and CD8^+^ T cells to the morphological and functional modifications occurring in astrocytes and their pathological outcomes.

## Introduction

Multiple sclerosis (MS) is a chronic inflammatory demyelinating disease of the central nervous system (CNS) affecting more than 2.5 million people worldwide, with a 2020 global prevalence of 35.9 per 100000 people (1). In the majority of patients, MS begins with a single clinically isolated syndrome (CIS) of neurological dysfunction that resolves over time. After the initial CIS, most patients have a second relapse and develop the relapsing-remitting MS (RRMS) form (2). Relapses are characterized by CNS inflammation and confluent area of demyelination in the white and grey matter of the brain and spinal cord caused by the loss of oligodendrocytes and myelin sheaths (3). One to two decades post-diagnosis, 15-30% of RRMS patients develop secondary progressive MS (SPMS) that is characterised by gradual neuroaxonal loss and brain atrophy, thus leading to patient disability and neurodegeneration (4). About 15% of patients develop an irreversible primary progressive form (PPMS) from the onset characterized by chronic demyelinated lesions in the white matter, axonal loss, diffuse and focal demyelination of the grey matter and neurodegeneration (5).

Despite the exact causes of MS remain still unknown, the disease is known to arise in genetically susceptible individuals (6, 7) by a complex interplay between environmental factors (8) and dysregulated immune responses (9). Several studies performed in murine models of experimental autoimmune encephalomyelitis (EAE) to explain MS pathophysiology, validated the hypothesis that MS is an autoimmune disorder characterized by the infiltration within the CNS of adaptive self-reactive immune cells, which cause demyelination and remyelination events, thus leading to the loss of sensory and motor functions (10). It is still an open question whether the initial MS triggering insult occurs within the CNS (intrinsic model), presumably affecting the oligodendrocytes and favouring the release of CNS antigens to the periphery, or whether it takes place outside the CNS (extrinsic model) leading to the activation of aberrant adaptive immune responses targeting CNS antigens (9). Independently of the place where the triggering events occur, peripheral innate and adaptive immune cells, especially autoreactive inflammatory T helper (Th) cells, cross the blood-brain barrier (BBB) and release inflammatory mediators in the brain that affect the function of resident glial cells, leading to astrogliosis, oligodendrocyte loss and axonal degeneration (11, 12).

Astrocytes are star-shaped glial cells that play a pivotal role in maintaining CNS homeostasis (13). Through highly ramified processes, astrocytes contact several cells within the CNS contributing to the formation, activity and plasticity of neuronal synapses (14), providing neurotrophic factors and metabolic support to neurons and oligodendrocytes (15–18) and ensuring the formation and maintenance of BBB integrity (19). At a resting state and in different brain regions, astrocytes are highly heterogeneous in their morphology and functional properties (20). Protoplasmic astrocytes, mainly located in the grey matter, at the hippocampus and cerebral cortex, are characterised by extremely ramified cell bodies, thus allowing them to contact synapses and perform neuromodulation. Fibrous astrocytes, mainly located in the white matter, are smaller with longer and narrower protrusions, which interact with axons at the level of the nodes of Ranvier (21). Besides these two main astrocyte subpopulations, nine more distinct astrocyte-like subtypes have been described on the basis of their morphological features including radial, marginal and perivascular glia located in the cortex of human brain (20). Moreover, the advent of single-cell RNA sequencing (scRNA-seq) and single-cell spatial transcriptomics evidenced further diversity and specialization of astrocytes depending on their differential brain localization (22–24).

In pathological conditions, such as MS, astrocytes undergo profound morphological and functional modifications (25, 26), which lead to a strong reduction of their metabolic and homeostatic functions (13, 27, 28). Moreover, astrocytes acquire a reactive phenotype characterized by the up-regulation of specific molecular markers such as glial fibrillary acidic protein (GFAP), vimentin, S100B, superoxide dismutase 1 (SOD1), complement component C3, tropomyosin receptor kinase B (TrkB) and IL-17R (29). In both MS and EAE animal models, inflammatory Th cells, once recruited to the CNS, produce cytokines such as TNF-α, IL-17, GM-CSF and IFN-γ (11) that activate astrocytes, which in turn acquire a reactive phenotype, proliferate, form glia scar (29, 30) and produce several cytokines and chemokines favouring the recruitment of leucocytes and inflammatory Th cells into the CNS parenchyma (25, 26, 31–34).

**Figure 1:**
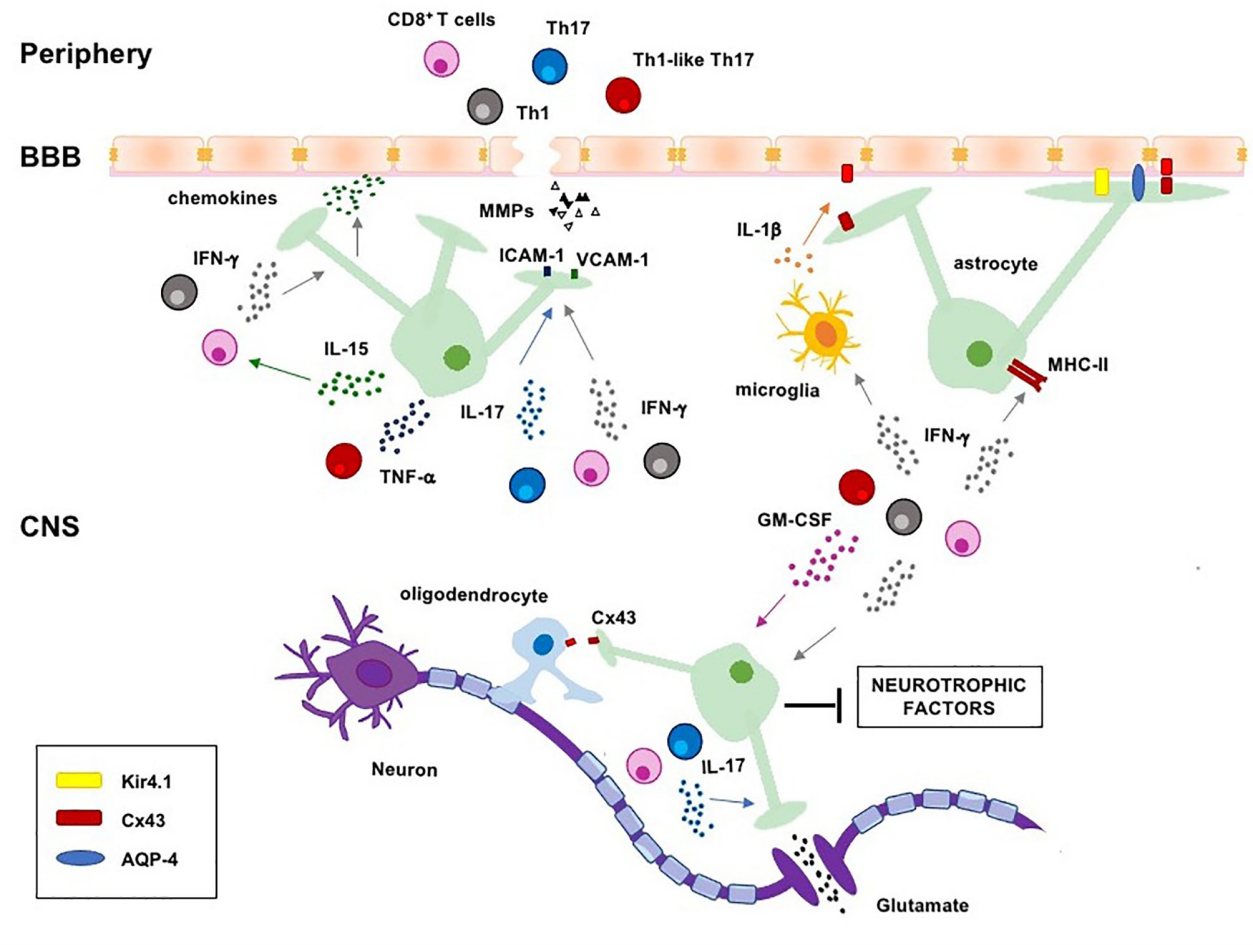
Regulation of astrocyte functions by pathogenic T cells and lineage-defining cytokines in MS. Pathogenic Th1, Th17, Th1-like Th17 and CD8^+^ T cells migrate into the CNS where they are reactivated and produce lineage-defining cytokines, which induce astrogliosis and negatively regulate several homeostatic functions of astrocytes, such as the maintenance of BBB integrity, clearance of excessive ions and glutamate from the synaptic cleft, energy support to neurons and oligodendrocytes. CNS, central nervous system; BBB, blood-brain-barrier; MHC-II, major histocompatibility complex II; ICAM-1, intercellular adhesion molecule-1; VCAM-1, vascular adhesion molecule-1; AQP-4, aquaporin 4; Cx43, connexin 43; Kir4.1, inward rectifying potassium channel 4.1.

In this review, we describe the main astrocytic changes occurring in MS and the role of the crosstalk between inflammatory T cells and astrocytes in amplifying CNS inflammation and MS progression (**Figure 1**).

## Astrocyte Dysregulation in MS

In both MS and EAE, the activation of astrocytes occurs at an early stage and persists into the acute and chronic stages of the disease. Several changes in both morphology and spatial localization of astrocytes have been observed in different stages of the disease (28). Studies in acute EAE showed the presence of hypertrophic reactive astrocytes at a very early stage of the symptomatic phase, even before immune cells cross the BBB and enter into the CNS parenchyma (35, 36). In acute MS lesions from post-mortem brain biopsies, hypertrophic reactive astrocytes with damaged end-feet processes were detected in active plaques as well as in the adjacent normal white and grey matter, thus suggesting their pivotal role in both the development and sustainment of the lesions (28, 37, 38). Astrocytes with a very swollen cytoplasm due to the accumulation of GFAP^+^ filaments were also found in active-acute lesions (28). Furthermore, the phenotypic characterization of reactive astrocytes in active demyelinating MS lesions evidenced that they lose most of their homeostatic functions and acquire a highly inflammatory and neurotoxic phenotype, thus inducing the death of both neurons and mature oligodendrocytes (39, 40). As recently evidenced by magnetic resonance imaging (MRI)-informed scRNAseq, astrocytes still remain active in chronic active demyelinated lesions and form an astroglial scar as soon as chronic lesions became inactive (41). Consistently, scRNAseq analysis of CNS samples from EAE mice identified a dominant cluster of high proinflammatory and neurotoxic astrocytes, characterized by increased GM-CSF signalling, NF-κB activation and iNOS expression. The presence of this reactive astrocyte subpopulation was also confirmed in post-mortem brain tissues from MS patients who underwent euthanasia followed by rapid autopsy (42).

### BBB Breakdown and Leukocyte Recruitment

The breakdown of the BBB is one crucial hallmark of MS (43, 44) that precedes the infiltration of peripheral leukocytes and autoreactive T lymphocytes that, once entered into the CNS, contribute to the development and expansion of MS lesions by damaging various cellular components of the BBB (45–50). The BBB is a continuous endothelial barrier between the CNS and peripheral blood that provides oxygen and critical nutrients to the CNS and limits the entry of toxic substances and immune cells. The integrity and functionality of the BBB are ensured by the physiological properties of highly specialized endothelial cells (EC) that, by interacting with pericytes, perivascular astrocytes and neurons, form a neurovascular unit (NVU) that limits both paracellular and transcellular movement of cells and solutes (51, 52). Perivascular astrocytes are crucial structural and functional components of the BBB that through their end-feet interact with ECs and ensheathe the brain vasculature (53). Disruption of the astroglia/NVU communication has been linked to BBB breakdown in both EAE and MS (37, 54). At the NVU, astrocytes produce several vasoactive molecules such as nitric oxide (NO), prostaglandins and arachidonic acid that regulate cerebral blood flow (55). Moreover, the specialized water-channel aquaporin-4 (AQP-4), the inward rectifying potassium channel (Kir) Kir4.1 and the gap junction-forming protein connexin 43 (Cx43) expressed by the end-feet at the level of glia limitans confer to astrocytes the ability to regulate the exchange of water and ions across the BBB (56). Extensive loss of Cx43 with concomitant patchy loss of AQP-4 was observed in actively demyelinating and chronic active lesions of progressive MS patients with high relapse rates (57), thus contributing to the weakened of BBB and to the vasogenic oedema due to increased hydrostatic vascular pressure and shear stress (**Figure 1**) (58).

The integrity of the BBB is also maintained by the tight junctions (TJs), large multiple transmembrane proteins containing occludins, claudins and junctional adhesion molecules (JAMs), which mediate tight adhesion between adjacent ECs. Claudins are the major components of BBB TJs and are essential for the maintenance of BBB integrity (19). The downregulation of claudin-5 and claudin-11 at the BBB has been associated with the impairment of barrier function (59, 60). In both MS and EAE lesions, reactive astrocytes upregulate thymidine phosphorylase (TYMP) and vascular endothelial growth factor A (VEGFA), which mediate the downregulation of occludin and claudin-5, thus contributing to BBB breakdown (61, 62). Furthermore, reactive astrocytes also produce CC-chemokine ligand 2 (CCL2), which contributes to the disassembly of TJs (63) and to the downregulation of both occludin and claudin-5 (64).

In addition to produce soluble factors that increase the permeability of the BBB, reactive astrocytes secrete several chemokines that favour the recruitment of circulating leukocytes into the CNS (**Figure 1**). CCL2 upregulation was observed in astrocytes from the white matter lesions of both MS post-mortem brains and EAE mice, where it plays a critical role in both macrophage and T cell infiltration into the white matter of spinal cord (31, 65, 66). In EAE and MS lesions, reactive astrocytes have been also identified as the major source of CCL20 (32, 33, 67), a chemokine that mediates the recruitment of pathogenic CCR6^+^ Th17 and Th1-like Th17 cells into the inflamed CNS (68). Furthermore, high levels of CXCL10 production by astrocytes correlated with the accumulation of CXCR3^+^ Th1 and Th1-like Th17 cells into the inflamed spinal cord and demyelinated lesions in EAE (25) (**Figure 1**).

### Impaired Astrocyte-Neuron Communication in MS

In the CNS, astrocytes are closely associated with neurons, by tightly enwrapping neuronal cell bodies, axons and synapses (**Figure 1**). The association between astrocytes and synapses is important to maintain the brain homeostasis and to regulate neuronal synaptic transmission (69). Astrocytes regulate synaptic functions by tuning glutamate concentration in the synaptic cleft (70). Glutamate is the major excitatory neurotransmitter in CNS that, if accumulates in the synaptic and extra-synaptic space, may lead to the hyperexcitation of neurons and neuronal death through a process known as glutamate excitotoxicity (71). After release from presynaptic neurons, glutamate is taken up from post-synaptic receptors such as mGluRs (metabotropic glutamate receptors), NMDARs (N-methyl-D-aspartate receptors) and AMPARs (α-amino-3-hydroxy-5-methyl-4-isoxazolepropionic acid receptors), which in turn transmit the excitatory impulse (72). Excessive and prolonged stimulation of glutamate receptors leads to the depolarization of postsynaptic membranes and mitochondrial Ca^2+^ overload that triggers excessive production of reactive oxygen species (ROS) and nitric oxide (NO) and the opening of the mitochondrial transition pore, thus favouring the release of pro-apoptotic proteins and neuronal cell death (73). Astrocytes play a crucial role in removing the excess of glutamate by using specific glutamate transporters such as glutamate-aspartate transporter (GLAST) and glutamate transporter-1 (GLT-1), which uptake more than 80% glutamate released in the synaptic cleft. In astrocytes, glutamate is then metabolized by glutamine synthase (GS) into glutamine that in turn is released, taken up by neurons and used for the synthesis of glutamate and gamma-aminobutyric acid (GABA) (71). Increased levels of glutamate were observed in the CSF of RRMS patients with active lesions during relapse as well as in SPMS patients (74). Moreover, in both MS and EAE, impaired glutamate homeostasis has been related to both decreased glutamine synthetase and increased glutaminase, the enzyme responsible for glutamate synthesis, as well as to the downregulation of the glutamate transporters GLT-1 and GLAST (75–79). In addition to impaired glutamate uptake, reactive astrocytes were also described to release glutamate at the peak stage of EAE by up-regulating the glutamate carboxypeptidase II, a metalloprotease that converts the neuropeptide N-acetylaspartylglutamate into N-acetyl aspartate and glutamate (80).

Astrocyte end-feet express several channels and ion transporters, which regulate ion homeostasis and neuronal excitability. In particular, Kir4.1 K^+^ channel, co-expressed together with AQP4 in the astrocytes end-feet, faces neuronal synapses and allows a rapid clearance of K^+^ ions from the extracellular space, thus facilitating the repolarization of neuronal membranes and neuronal firing (81). Reduced levels of Kir4.1 were observed in perivascular astrocytes in both acute and chronic active demyelinated MS lesions (82). The reduction of astroglial Kir4.1 channel was associated to increased serum levels of complement-fixing IgG subclasses, suggesting a role of anti-Kir4.1 autoantibody in amplifying inflammation and tissue damage in MS (82, 83).

Another important function of astrocytes is to regulate the brain energy supply required for the transmission of synaptic impulses and neuron functions (18). Astrocytic end-feet rapidly uptake glucose from the brain capillaries and metabolize it in the glycolytic route to generate lactate. Lactate is then transferred through monocarboxylate transporters (MCT) to neurons where it is converted to pyruvate and oxidized for energy production in mitochondria (84). Interestingly, a significant reduction in the expression of genes encoding for both the astrocyte-neuro lactate shuttle (ANLS), including MCT1, and the glutamate–glutamine cycle (GGC), including glutamine synthase and GLT-1, has been observed in the grey matter from post-mortem brain tissues of chronic SPMS and PPMS patients (85). The downregulation of both ANLS and GGC genes observed in MS was also associated with the simultaneous up-regulation of inflammatory cytokines suggesting a role of immune-related signalling in the impairment of astrocyte metabolic functions (85). Consistently, Ponath et al. found that astrocytes derived from pluripotent stem cells of MS patients carrying the risk allele variant rs7665090^G^ produced large amounts of proinflammatory factors and displayed a significant reduced ability to release lactate and reuptake glutamate after stimulation (86). The further analysis of reactive astrocytes in white matter lesions from post-mortem tissues suggested the presence of harmful hypertrophic astrocytes in MS patients carrying the risk allele variant (86). The impaired metabolic functions of astrocytes observed in MS also involve the synthesis of cholesterol that is required for myelin sheath formation, the maintenance of axonal membrane and synapses integrity (87). Astrocytes from spinal cord, cerebellum and optic nerve of chronic EAE showed a reduction in the expression of several genes involved in cholesterol synthesis. Similar results were observed by the gene expression analyses of astrocytes from optic chiasm autopsy tissues from MS patients (88).

In order to support the correct neuronal effector responses, astrocytes also secrete neurotrophic factors including nerve growth factor (NGF), brain-derived neurotropic factors (BDNF), fibroblast growth factor (FGF) and ciliary neurotrophic factor (CNTF), which are required for the optimal survival, growth and differentiation of neurons and for preventing neurodegeneration (89). Impaired production of neurotrophic factors was observed in astrocytes exposed to T cell-derived inflammatory cytokines in EAE (90). Moreover, in both EAE and chronic MS lesions, astrocytes up-regulated the BDNF receptor TrkB that upon stimulation with BDNF induced a strong release of NO, thus contributing to oxidative stress and neuronal damage (91).

### Impaired Astrocyte-Oligodendrocyte Communication in MS

Reactive and hypertrophic astrocytes, accumulating in MS demyelinating lesions, also contribute to oligodendrocyte loss and demyelination by favouring lesion development and progression (38, 92). In healthy brains, astrocytes regulate the homeostasis of myelin sheaths by releasing several neurotrophic factors that promote the proliferation of oligodendrocyte progenitor cells (OPCs) their migration and differentiation to oligodendrocytes (93). The platelet-derived growth factor (PDGF) and BDNF secreted by astrocytes promote OPCs proliferation, migration and maturation to myelinating oligodendrocytes (94). During neuroinflammation, astrocytes also exert neuroprotective effects on oligodendrocytes by producing CXCL1 and CNTF, which favour OPCs recruitment to axons and their differentiation into mature myelinating oligodendrocytes, respectively (95). Consistently, reactive astrocytes in acute MS lesions secrete several remyelinating factors (96) and increased BDNF release by reactive astrocytes was found to induce remyelination in a cuprizone-induced demyelination model (97). However, with the progression of MS to a chronic stage, reactive astrocytes form a dense glial scar around the axons and secrete hyaluronan and proteoglycans, thus preventing OPCs recruitment and maturation into the demyelinated areas (97, 98). Moreover, astrocytes also supply lipids, especially cholesterol, to oligodendrocytes necessary for myelin synthesis (16, 99). This astrocyte-oligodendrocyte network is finely regulated by a physical interaction through connexins Cx30/Cx32 and Cx43/Cx47, which are fundamental for the exchange of potassium ions and metabolic factors required for myelin maintenance (16, 100, 101). A strong reduction of Cx47 in both cell bodies and proximal oligodendrocyte processes as well as of the astrocyte binding partner Cx43 was observed in EAE lesions. A concomitant loss of Cx32 was also detected within and around the lesions that persisted throughout the disease course (102). Similar results were obtained by immunohistochemical analysis of post-mortem brain tissues from MS patients, where a strong reduction of both oligodendrocyte Cx32 and Cx47 was observed in and around chronic lesions as well as in the normal-appearing white matter (NAWM) (103). On the contrary, the expression of Cx30 and Cx43 on astrocytes was increased in both lesions and NAWM and correlated with astrogliosis and the acquisition by astrocytes of an inflammatory phenotype, while higher Cx32 expression was associated with a longer disease duration (104). So, the loss of connection between oligodendrocytes and reactive astrocytes during chronic inflammation may accelerate MS progression by contributing to demyelination and axonal damage. Consistently, Cx43 loss was associated with a rapidly progressive MS course, oligodendrogliopathy and active demyelinating lesions (57).

## Dysregulation of Astrocyte Functions by Inflammatory T Cells in MS

The immunopathogenesis of MS relies on the recruitment of specific autoreactive Th cell subsets and CD8^+^ T cells within CNS where they are reactivated and secrete cytokines and chemokines that modulate the activity of several glial cells, including astrocytes (11, 105). Among Th cells, Th1, Th17 and Th1-like Th17 cells have been identified as key players of MS pathogenesis, by producing one or more lineage-defining cytokines, which affect several astrocyte functions as discussed below (**Figure 1**).

### Th1-Mediated Regulation of Astrocytes in MS

Th1 cells are a subset of CD4^+^ T lymphocytes characterized by the expression of the CXC chemokine receptor type 3 (CXCR3), interleukin (IL)-12 receptor (IL-12R) chains β1/β2, the master transcription factor T-bet and by the production of the lineage-signature cytokine IFN-γ together with GM-CSF and TNF-α (106). The neuropathological functions of Th1 cells in MS have been extensively studied in EAE animal models (11) and associated to their ability to trigger the activation of resident microglia and their differentiation into a high inflammatory and neurotoxic phenotype (107, 108). More recent studies evidenced that Th1 cells and their effector cytokines may also affect the phenotype and functions of astrocytes in MS (109). Human astrocytes, indeed, were found to express IFN-γ receptor (IFNGR) that was also up-regulated in the cortex of post-mortem MS brain tissues and associated with the acquisition of a neurotoxic phenotype (110). Silencing of IFN-γ signalling in murine astrocytes, suppressed EAE by inhibiting inflammatory chemokine production and the infiltration of Th1 and Th17 cells into the CNS (111, 112). Moreover, IFN-γ-treated astrocytes up-regulated the expression of chemokines such as CCL20, CXCL10 and CXCL12 involved in the recruitment of both Th1 and Th17 cells into the CNS, and CCL2 (90, 113) that contributes to BBB breakdown by inducing both disassembly and downregulation of TJs (63, 64). In addition to favour the infiltration of inflammatory T cells within CNS, IFN-γ-activated astrocytes were also described to promote the proliferation of myelin-specific T cells during EAE by up-regulating major histocompatibility complex class II (MHC-II) molecules and contributing to the reactivation of pathogenic T lymphocytes as antigen-presenting cells (APC) (114). Interestingly, astrocytes in chronic active lesions from post-mortem MS brain tissues were found to express MHC-II together with B7.1 and B7.2 (115, 116), two important costimulatory molecules that are required for optimal APC functions and up-regulated by IFN-γ (117, 118). However, IFN-γ was also described to mediate protective effects on astrocytes during chronic EAE. Smith et al. observed an exacerbation of chronic EAE, an increase of the lesion size and enhanced oxidative stress, in mice with IFNGR-deficient astrocytes (119). Similar results were obtained in EAE transgenic mice expressing a signalling deficient dominant negative IFNGR1 on astrocytes (120).

Th1-derived cytokines have been also implicated in polarizing astrocytes to a neurotoxic phenotype. In astrocytes from EAE mice, Th1-derived cytokines such as IFN-γ and GM-CSF impaired the expression of neurotrophic factors such as NGF, CNTF and BDNF, and up-regulated the expression of NO synthase (90). Moreover, the loss of Cx43 in astrocytes from acute demyelinating MS lesions has been recently associated to Th1-derived IFN-γ *via* microglia-dependent production of IL-1β, thus contributing to the disruption of astrocyte intercellular communications and MS progression (121).

### Th17-Mediated Regulation of Astrocytes in MS

Th17 cells are characterized by the expression of CCR6, CCR4, CD161, IL-23R, IL-1R, the master transcription factor retinoic acid receptor-related orphan nuclear receptor γt (RORγt) and the production of the lineage-signature cytokines IL-17A-F and IL-21 together with IL-22 (122). The pathogenic functions of Th17 cells in MS have been associated to BBB breakdown and CNS inflammation (123, 124) by targeting both resident microglia and astrocytes (90, 107). Astrocytes, indeed, express a functional IL-17R and are responsive to IL-17 by polarizing towards a reactive phenotype and by producing several inflammatory cytokines and chemokines during EAE (67, 125–127). The impairment of IL-17-mediated signalling in astrocytes through the selective ablation of key signalling mediators was shown to ameliorate EAE by inhibiting the production of inflammatory chemokines, the infiltration of inflammatory cells (128, 129) and the percentage of Th17 cells within CNS (130). For instance, in astrocytes, IL-17 induces the expression and production of IL-6 that, by acting in a positive feedback loop, may amplify Th17 cell differentiation (131–133). IL-17 also enhances the production of CCL20 in astrocytes (67, 128), thus facilitating the recruitment of Th17 cells within CNS (90). Moreover, by up-regulating the expression of vascular adhesion molecule-1 (VCAM-1) on brain stem astrocytes, Th17-associated cytokines may further enhance the recruitment of both Th1 and Th17 cells within the CNS (134).

IL-17-mediated signalling in astrocytes also promotes the secretion of matrix metalloproteinases such as MMP-3 and MMP-9 that further compromise the integrity of the BBB favouring the recruitment of encephalitogenic T cells into the CNS (129, 130).

Th17 cell-associated cytokines were also shown to affect the homeostatic functions of astrocytes. Kostic et al. reported that low doses of IL-17A impaired the ability of astrocytes to uptake glutamate from the extracellular space by reducing the expression of GLT-1 and GLAST transporters as well as of glutamine synthetase. In addition to reduce glutamate uptake, exposure of astrocytes to IL-17A also caused a Ca^2+^-dependent glutamate release, thus favouring excitotoxic damage (**Figure 1**) (135).

### Role of Th1-Like Th17 Cells on Astrocyte Functions in MS

Despite most of the studies carried out to investigate the crosstalk between inflammatory Th cells and astrocytes focused on Th1 and Th17 cells, the recent identification of highly pathogenic Th1-like Th17 cells in both EAE (49, 136) and MS (136–140) suggests their contribution in promoting the morphological and functional changes occurring during astrogliosis. Th1-like Th17 produce TNF-α, GM-CSF, IL-17A, although at lower levels than Th17 cells, high levels of IFN-γ, co-express CXCR3 and T-bet together with CCR6 and RORγt, and express IL-23R (122, 141, 142).

Most of the cytokines produced by Th1-like Th17 cells may exhibit synergistic detrimental effects on astrocytes. For instance, IFN-γ and TNF-α cooperate with IL-17A by inducing the production of inflammatory chemokines in astrocytes and enhancing the recruitment of encephalitogenic T cells into the CNS (90, 128, 132, 143). Moreover, IFN-γ and IL-17A produced by Th1-like Th17 cells induce the up-regulation of intercellular adhesion molecule-1 (ICAM-1) and VCAM-1 in cortical astrocytes within CNS lesions during EAE (144). Finally, a detrimental contribution of Th1-like Th17 cells on astrocytes is strongly supported by the above cited effects of IFN-γ in combination with GM-CSF. Indeed, these cytokines cooperate in impairing the production of neurotrophic factors as well as in promoting oxidative stress in astrocytes (90) and enhance astrocyte-dependent glutamate excitotoxicity induced by IL-17A (**Figure 1**) (135). Nevertheless, further studies are required to elucidate this issue.

### Crosstalk Between CD8^+^ T Cells and Astrocytes in MS

Although inflammatory Th cell subsets have long been regarded as the main effectors of MS pathogenesis, an important pathophysiological role of CD8^+^ T cells has also recently been recognized. Histopathological studies of immune cell infiltrates in post-mortem brain tissues from MS patients showed a prevalence of CD8^+^ T cells compared to CD4^+^ T cells (105, 145). Moreover, sRNA-seq analyses revealed a prominent oligoclonal expansion of CD8^+^ T cells in the peripheral blood and CSF of MS patients (146, 147).

Activated CD8^+^ T cells may contribute to BBB breakdown by inducing the activation of astrocytes and the downregulation of both occludin and claudin-5 in perforin-dependent and non-apoptotic manner (148). Once entered into the CNS, CD8^+^ T cells may be reactivated by MHC class I-expressing resident glial cells, including reactive astrocytes (145), thus contributing to tissue damage and neuroinflammation (149). In EAE, CD8^+^ T cells also produce IL-17 thus supporting Th17-mediated dysregulation of astrocyte functions (150). Astrocytes in turn may enhance the cytotoxic activity of CNS-infiltrating CD8^+^ T cells by producing IL-15 (151). Immunohistochemistry analysis of post-mortem brain tissues from MS patients revealed the expression of IL-15 in reactive GFAP^+^ astrocytes located in both acute and subacute MS lesions as well as near blood vessels. The exposure of CD8^+^ T cells to astrocyte-derived IL-15 enhanced antigen-specific cytotoxicity as well as the expression of lytic enzymes (granzyme B and perforin) and natural killer group 2 member D (NKG2D) (151). NKG2D is a transmembrane receptor constitutively expressed on human CD8^+^ T cells that recognizes stress-induced ligands on target cells and enhances TCR-mediated cytotoxicity (152). Notably, ULBP4, a NKG2D ligand, was highly expressed on astrocytes in active and chronic active MS lesions from post-mortem brain tissues. Furthermore, the addition of soluble ULPB4 to CD8^+^ T cells from MS patients co-cultured with astrocytes enhanced the production of inflammatory cytokines and increased T cell motility, thus suggesting an important contribution of the crosstalk between CD8^+^ T cells and astrocytes in synergy with CD4^+^ Th cells to CNS damage in MS (153).

### Impact of T Cell Targeting MS Therapeutic Drugs on Astrocytes

The crosstalk between inflammatory T cells and astrocytes in sustaining neuroinflammation and neurodegeneration in MS is supported by recent evidences that some of the disease-modifying drugs target both inflammatory T cells (11) and astrocytes (154).

Glatiramer acetate (GA), an acetate salts composed of a mixture of four synthetic polypeptides (glutamate, lysine, alanine, and tyrosine), suppresses inflammatory Th1 cells by promoting their shift to an anti-inflammatory Th2 cell phenotype (155–157) and by increasing the frequency of suppressive regulatory T cells (Treg) (158). In EAE mice, GA-specific Th2 cells produced high levels of BDNF, IL-10 and TGF-β following their adoptive transfer within CNS (159). Moreover, the production of immunosuppressive cytokines and neurotrophic factors by GA-specific T cells primed astrocytes to produce IL-10 and TGF-β (159), thus promoting their transition from a neurotoxic to a neuroprotective phenotype (160). More recent data from Eilam et al. also evidenced that GA treatment of EAE mice partially abrogated BBB disruption by increasing the expression of claudin-5 on astrocytes and by restoring their end-feet connections with the NVU (54).

Dimethyl fumarate (DMF) acts on T cells by reducing the total number of circulating T cells (161, 162), in particular of IFN-γ- and IL-17-producing Th cell subsets and CD8^+^ T cells (163, 164), and by increasing the percentage of Th2 and Treg cells (165, 166). DMF treatment of primary reactive astrocytes derived from both human and murine brains evidenced the ability of this drug to reduce the secretion of inflammatory cytokines and chemokines as well as to prevent the production of ROS (167) by promoting anti-oxidant gene expression (168). Similar effects were observed by treating human astrocytes with a novel fumarate, isosorbide-DMF (IDMF). Genome-wide expression analysis of human astrocytes treated with DMF and IDMF evidenced the ability of both compounds to downregulate the expression of several genes associated with neurotoxic reactive astrocytes, including MMP9, CCL2 and ICAM1 (169).

Fingolimod (FTY720) and siponimod (BAF312) are two antagonists of the sphingosine-1-phosphate receptor (S1PR) approved for RRMS and SPMS, respectively (11, 170). Both drugs bind S1PR and induce its internalization, thus sequestering T cells in lymphoid organs and reducing circulating inflammatory CD4^+^ and CD8^+^ T cells (11, 170–172). In both human and murine astrocytes, fingolimod treatment was shown to impair the production of inflammatory and neurotoxic factors (173) and to promote the secretion of neurotrophic mediators (174). Moreover, Trkov Bobnar et al. showed that fingolimod reduced the expression of MHC-II on the surface of IFN-γ-treated astrocytes, thus preventing their activity as APCs (175). As fingolimod, siponimod was reported to impair inflammatory cytokine expression in human astrocytes and to restore astrocyte-endothelial cell connections by up-regulating the expression of claudin-5 and ZO-1 (176, 177). Finally, in human astrocytes, both fingolimod and siponimod prevented glutamate neurotoxicity by restoring the expression of GLAST and GLT1 on astrocytes and glutamate uptake (177, 178).

Laquinimod (LQ) is a quinoline-3-carboxamide derivate under clinical trial evaluation for the treatment of RRMS (179). In EAE, LQ treatment ameliorated disease progression by reducing the polarization and recruitment of Th17 cells as well as the production of inflammatory cytokines (180, 181). Treatment of human astrocytes with LQ inhibited IL-1β-induced downregulation of glutamate transporters GLAST and GLT1 and restored astrocyte glutamate uptake (178). Consistently, LQ treatment ameliorated EAE by suppressing, in astrocytes, the expression of inflammatory mediators such as IL-6 and ROS and by inducing a transcriptional program associated to homeostatic chemokines, neurotrophin, axonal guidance and transendothelial migration (182).

## Conclusions

The contribution of Th1, Th17, Th1-like Th17 inflammatory cytokines in activating astrocytes to gain a neurotoxic phenotype is beginning to be studied in MS. However, astrocytes may also counteract inflammation by producing several factors, which reduce BBB breakdown, leucocyte infiltration and promote remyelination, axonal regeneration and neurogenesis (21). Therefore, therapeutic strategies aimed at counteracting the infiltration of inflammatory Th cell subsets into the CNS and at polarizing reactive astrocytes towards a neuroprotective phenotype may be beneficial to arrest disease progression as well as to stimulate repair processes. A deeper understanding of the cross-talk between T cells and astrocytes in MS will be seminal for the development of more efficient therapies dampening inflammatory T cells and stimulating neuroprotective astrocytes.

## Author Contributions

MK, CA, and VT wrote the original draft. MF contributed to reviewing and editing the manuscript. LT contributed to writing and editing the manuscript. All authors contributed to the article and approved the submitted version.

## Funding

This work was supported by the Italian Foundation for Multiple Sclerosis (FISM 2020-R-Single/01), “Progetto Ateneo” (Sapienza University of Rome, Italy) and Istituto Pasteur Italia-Fondazione Cenci Bolognetti (Sapienza University of Rome, Italy) to LT. Fondazione Ceschina (Lugano, Switzerland) to MF.

## Conflict of Interest

The authors declare that the research was conducted in the absence of any commercial or financial relationships that could be construed as a potential conflict of interest.

The handling editor declared a past co-authorship with the authors MK, CA, and LT.

## Publisher’s Note

All claims expressed in this article are solely those of the authors and do not necessarily represent those of their affiliated organizations, or those of the publisher, the editors and the reviewers. Any product that may be evaluated in this article, or claim that may be made by its manufacturer, is not guaranteed or endorsed by the publisher.
